# Blood Flow Restriction Training for Improving Body Composition of a 26-Year-Old Male With L5-S1 Disc Protrusion and Nerve Root Compression: A Case Report

**DOI:** 10.7759/cureus.70055

**Published:** 2024-09-23

**Authors:** Sneha Thirugnana Sambandam, Dobson Dominic, Senthuran Selvam, Nitesh K Rathi

**Affiliations:** 1 Sports Medicine, Saveetha Medical College and Hospitals, Saveetha Institute of Medical and Technical Sciences, Chennai, IND; 2 Orthopaedics, Saveetha Medical College and Hospitals, Saveetha Institute of Medical and Technical Sciences, Chennai, IND

**Keywords:** bfr, kaatsu training, low back pain, occlusion training, spine injury, spine rehabilitation

## Abstract

This case report explores the use of blood flow restriction (BFR) training to improve body composition in a 26-year-old male with L5-S1 disc protrusion and nerve root compression. BFR training, involving low-intensity exercises with restricted blood flow, offers a promising alternative for patients unable to engage in high-intensity workouts. The patient, a recreational gymgoer with a history of a significant lower back injury from a maximal deadlift event 20 months ago, presented with chronic pain, weight gain, and decreased stamina. Two attempts at spinal steroid injections during the 20-month period yielded only temporary relief, prompting the implementation of a 12-week BFR training regimen. The program combined BFR exercises with a calorie-deficit diet, resulting in substantial improvements in body composition and strength. Over 12 weeks, the patient lost 11.68 kg, reduced his body mass index from 26.50 to 22.85, and decreased his total body fat percentage from 28% to 22.43%. His lower back pain also significantly improved. This case highlights the effectiveness of BFR training in managing obesity and enhancing physical fitness in spinal injury patients, emphasizing the need for further research on its broader application.

## Introduction

Blood flow restriction (BFR) training, also known as Kaatsu or vascular occlusion training, is an upcoming exercise modality that involves exercising with the application of external pressure in the proximal part of limbs using specialized devices that restrict blood flow. Equipment used to exert pressure includes inflatable cuffs, elastic bandages, compression wraps (such as blood pressure cuffs), or other restraint bands specifically designed for BFR training [1]. The bands exert sufficient pressure to block arterial flow while occluding venous flow partially [1]. BFR training has various applications. Research has shown that BFR training combined with low-intensity resistance training can increase muscle mass and strength, while BFR training combined with endurance training can improve cardiovascular fitness levels [2].

BFR training is superior to traditional training practices as it is less load-bearing, which generates less mechanical strain while it induces greater metabolic stress [3]. The possible mechanisms through which BFR training produces various effects are increases in hormone concentrations, increases in intracellular signaling pathways for muscle protein synthesis, such as the mechanistic Target of Rapamycin (mTOR) pathway, increases in biomarkers indicating satellite cell activity, and apparent patterns in fiber type recruitment [1]. There have also been scientific studies that show hypertrophic effects in both BFR limbs and non-BFR muscles during BFR training programs [4].

Limited research has been conducted on using BFR training in the patient population. A review and meta-analysis in the year 2017 on BFR training in clinical musculoskeletal rehabilitation showed that only 20 studies evaluated the effect of BFR training on strength, and the effect size was found to be moderate [5]. Since then, there have been a limited number of case reports and randomized control trials on patients with knee osteoarthritis/injuries, rotator cuff tendinopathy, lateral elbow tendinopathy, extremities fracture, and patients post anterior cruciate ligament rehabilitation [6-10].

For spinal injury patients, maintaining muscle mass and improving body composition is crucial yet challenging due to the limited ability to perform high-intensity exercise and reduced mobility. BFR training is a viable option to implement as it improves strength even while training at low intensities, thereby reducing the risk of re-injury [1]. The primary benefit of BFR training is its ability to produce muscular hypertrophy and strength increases comparable to those obtained by high-intensity training but with considerably lighter weights, often 20-30% of one's one-repetition maximum (1RM) [1].

BFR training has gained attention in neurological rehabilitation for its potential to improve strength and muscle mass in individuals with nerve injuries or conditions affecting the nervous system [11,12]. BFR involves restricting blood flow to muscles during low-intensity exercises. This is particularly beneficial in neurological rehabilitation, where patients often face reduced mobility and the inability to engage in high-load exercises. Studies have shown that BFR can stimulate neuromuscular adaptations even in patients with spinal cord injuries, aiding in muscle preservation and functional recovery [11,12]. The cross-transfer effect, where training one limb with BFR can improve strength in other non-restricted limbs, further highlights its usefulness in treating unilateral neurological deficits [11].

However, the specific effects of BFR training on body composition in spinal injury patients remain largely unexplored. This case study aims to bridge the research gap by utilizing this novel approach to evaluate its feasibility and effect on the body composition of a young male with L5-S1 disc protrusion with nerve root compression.

## Case presentation

A 26-year-old recreational gym goer presented to the Sports Medicine outpatient department (OPD) of Saveetha Medical College and Hospitals with concerns about body composition. In particular, he was seeking to reduce body fat and increase muscle mass. His primary goal was to improve body composition and regain fitness. He sustained trauma to the lower back two years ago while performing a maximal deadlift event during a power-lifting competition. This incident resulted in a right paracentral disc protrusion at the L5-S1 intervertebral disc, causing lateral recess narrowing with compression over the ipsilateral traversing nerve root, as seen on magnetic resonance imaging (MRI).

**Figure 1:**
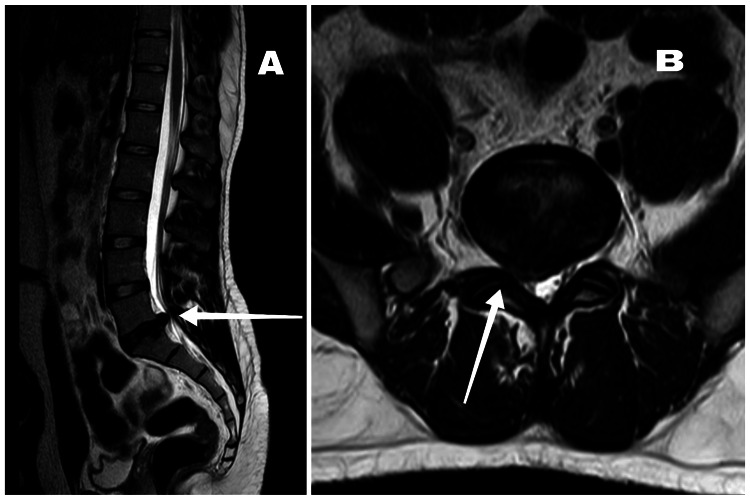
(A) Sagittal T2-weighted magnetic resonance image of lumbar spine. (B) Axial T2-weighted magnetic resonance image of spine at L5-S1 level. The arrow in image A points at the disc protrusion at L5-S1 level. The arrow in image B points at the right paracentral disc protrusion at L5-S1 level, indenting the anterior thecal sac causing significant narrowing of the spinal canal and right lateral recess.

Following the injury, he experienced chronic low back pain, which radiated to his right lower leg. He also gives a history of numbness and tingling sensation in the same leg. Four months after the injury sustained during a deadlift, he received an epidural steroid injection, which provided temporary relief, and he was advised to rest for a few weeks. Four months post the injection, he experienced pain again with radiating symptoms. He tried conservative management for two months after the injection with exercise therapy and pain medications. Since the pain did not subside, the MRI was repeated, which revealed right paracentral disc protrusion at L5-S1, indenting the anterior thecal sac, causing significant narrowing of the spinal canal and right lateral recess and compressing the right traversing nerve root and abutting the right exiting nerve root (Figure 1). He underwent an image-guided nerve root block at the right L5 nerve post, which pain subsided, and he was once again advised relative rest for two days, after which he underwent a spinal rehabilitation program for four months.

The patient presented to the OPD six months after the completion of the rehabilitation program (20 months after injury), seeking advice regarding body composition. He complained of mild intermittent lower back pain, which aggravated on heavy lifting and prolonged sitting, an increase in body weight, and a decrease in stamina. He gives a history of weight gain of 15 kg in the last 20 months. He used to follow a strength and conditioning program prior to the injury at a local gym but discontinued after the injury. His spinal rehabilitation program included light-intensity resistance training with minimal aerobic training.

On examination, he appeared to be a well-nourished, overweight individual. His vitals were within normal limits, and systemic examination revealed no abnormalities. A local examination of the lumbar spine showed that he had tenderness over the L5-S1 region and minimal pain on lumbar flexion. His height was 179 cm, weight was 84.88 kg, and his body mass index (BMI) was estimated to be 26.50 kg/m^2^. Skin fold calipers were used to estimate this total body fat percentage (TBFP) using the Jackson-Pollock seven-site method as 28%. His waist-hip ratio (WHR) was 0.98.

**Figure 2:**
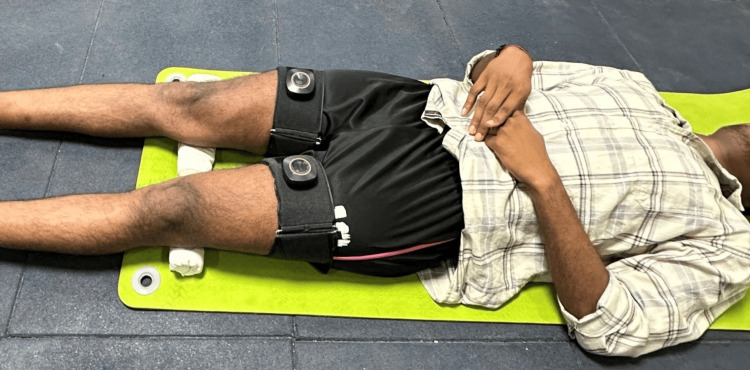
The patient performing lower limb compound exercises with the blood flow restriction (BFR) band (VALD AirBands, VALD Performance Brisbane, Australia) applied at the proximal part of the lower limb.

After obtaining consent from the patient, a holistic approach was undertaken to manage his obesity with a strength and conditioning plan using BFR training, a calorie deficit diet, and a good sleep and hydration plan. He was advised on a 12-week exercise program using BFR training. His program was planned in such a way that it included whole body light to moderate intensity exercise that did not cause or aggravate his lower back pain. The equipment used to execute the BFR training was AirBands, which is produced by an Australian-based company called VALD Performance (Brisbane, Australia) (Figure 2). His detailed exercise program is given in Table 1. The diet prescribed was high protein in nature, and he was at a deficit of 500 calories per day. The diet was curated by a certified nutritionist. He was further advised seven to eight hours of sleep per day and asked to consume 3 to 4 L of water per day.

**Table 1:** Twelve-week exercise prescription Training goal - Improve the strength of the upper limb, lower limb, and core while further strengthening the spine. Overall exercise plan - Alternate sessions of the upper limb and lower limb-focused training day. BFR equipment - AirBands (VALD Performance, Brisbane, Australia). Weight prescription - 1RM was assessed at the beginning and every four weeks, and weights were prescribed accordingly. Compound exercises - Utilization of several muscle groups with multi-joint movement to perform an exercise. Isolation exercise - Utilization of a single group with single-joint movement to perform an exercise. AOP, arterial occlusion pressure; BFR, blood flow restriction; reps, repetitions; 1RM, one repetition maximum

Training period	Exercise prescription	Training duration	Training intensity	Training volume
Weeks 1 to 4 (three to four sessions per week)	Warmup (whole body exercise without aerobic training)	30 to 45 minutes of training including five to 10 minutes of BFR training	BFR - upper limb with 30-40% AOP at 20-30% 1RM	BFR - compound exercises (total 75 reps) 30 × 15 × 15 × 15 or sets to failure 30-60 seconds rest between each set
	Two compound exercises - BFR training		BFR - lower limb with 50-60% AOP at 20-30% 1RM	Isolation (total 45 reps) 15 × 15 × 15 30-45 seconds rest between each set
	Two isolation exercises		Isolation exercises of upper limb and lower limb at 40-60% 1RM	Core and spinal exercises (timed or repetitions as tolerated)
	Five core with spinal strengthening exercises			
Weeks 5 to 8 (four to five sessions per week)	Warmup - aerobic training (cycling/treadmill) - 10 minutes	40 to 50 minutes of training including five to 10 minutes of BFR training	BFR - upper limb with 40-50% AOP at 20-30% 1RM	BFR - compound exercises (total 75 reps) 30 × 15 × 15 × 15 or sets to failure 30-60 seconds rest between each set
	Two compound exercises - BFR training		BFR - lower limb with 60-70% AOP at 20-30% 1RM	Isolation (total 50 reps) 12 × 15 × 15 30-45 seconds rest between each set
	Two isolation exercises		Isolation exercises of upper limb and lower limb at 40-60% 1RM	Core and spinal (timed or repetitions as tolerated)
	Five core with spinal strengthening exercises			
Weeks 8 to 12 (four to five sessions per week)	Warmup - aerobic training (cycling/treadmill) - 15 minutes	45 to 60 minutes of training including five to 10 minutes of BFR training	BFR - upper limb with 50-60% AOP at 30-40% 1RM	BFR - compound exercises (total 75 reps) 30 × 15 × 15 × 15 or sets to failure 30-60 seconds rest between each set
	Three compound exercises - BFR training		BFR - lower limb with 70-80% AOP at 20-30% 1RM	Isolation (total 60 reps) 30 × 15 × 15 30-45 seconds rest between each set
	Two isolation exercises		Isolation exercises of upper limb and lower limb at 40-60% 1RM	Core and spinal (timed or repetitions as tolerated)
	Five core with spinal strengthening exercises			

His weight, BMI, TBFP, and WHR were checked periodically at four-week intervals. His lower back pain significantly improved over the 12-week period. He lost 11.68 kg, and his BMI reduced to 22.85, while his TBFP and WHR also decreased post the 12-week intervention (Table 2). No adverse events occurred during the course of the treatment, and the patient was compliant with all the exercises.

**Table 2:** Periodic assessment of body composition of the patient BMI, body mass index; cm, centimeters; kg, kilograms; kg/m², kilograms/meter squared; TBFP, total body fat percentage; WHR, waist-hip ratio

Time of assessment	Height (cm)	Weight (kg)	BMI (kg/m^2^)	TBFP	WHR
Baseline	179	84.88	26.50	28	0.98
End of four weeks	179	81.37	25.40	26.32	0.96
End of eight weeks	179	78.64	24.54	24.17	0.93
End of 12 weeks	179	73.20	22.85	22.43	0.89

The patient’s 1RM of all exercises also increased during the course of the exercise program. The patient gave feedback that the perceived exertion during BFR training was manageable and less intimidating than doing heavy resistance training. He also expressed high satisfaction with the program and gave feedback that he was able to adhere to the prescribed exercises without difficulty.

## Discussion

This case study presents the journey of a 26-year-old with L5-S1 disc protrusion with nerve root compression from spinal injury to physical deconditioning to eventual return to fitness. The patient’s history highlights the significant impact of an acute injury on long-term physical health. The case study shows that BFR training is a feasible and effective treatment modality in improving body composition in a spinal injury patient. Not only the patient’s body composition but also his 1RM improved over the course of the treatment, emphasizing the effectiveness of BFR training in improving composition and improving strength. It further sheds light on taking a multidisciplinary approach to bringing about lifestyle modifications for the betterment of health.

The L5-S1 disc is a common site for disc herniation due to the high mechanical stress it endures [13]. During deadlift, increased load is sustained in the lower back. The exercise likely caused an increase in intradiscal pressure, leading to protrusion [14]. The possible mechanism through the initial management with intraspinal steroids would have reduced pain by reducing inflammation [15]. However, the treatment may not have addressed the underlying biomechanical issues leading to rebound pain. Studies have shown that though intraspinal steroid injections help mitigate pain in the short term, their long-term effects are debatable [15].

The rest period advised post the injection may have been beneficial in terms of reducing pain and inflammation but led to significant deconditioning. Prolonged inactivity can lead to a significant decrease in an individual’s cardiovascular fitness and strength, as well as an increase in fat, which were all observed in this patient. The weight gain and loss of fitness future compounded to the patient’s challenges as increased body weight can exacerbate mechanical stress on the spine, potentially worsening the symptoms of disc protrusion and nerve compression. The second steroid injection may have had similar effects to the previous treatment but may have yielded better results due to targeted treatment.

When the patient presented to the OPD, his BMI fell under the obesity grade 1 category according to the World Health Organization (WHO) classification, but after the treatment, his BMI fell under the normal category [16]. The results show that BFR training, combined with a calorie-deficit diet and regular physical exercise, helps improve body composition. A previous study conducted by Herda et al. produced contrasting results [17]. The study found that low-volume and intensity walking with BFR did not alter body composition in a group of highly active adults [18]. However, the study’s population was previously active individuals, and the study program did not include resistance training. Hence, BFR training may not have had an effect on body composition [18]. In a group of recreational runners, BFR training elicited a positive effect on body composition; the results of the study parallel the results of this study. 

A case report by Krogh et al. demonstrated the feasibility and safety of a four-week blood flow-restricted exercise program in an individual with tetraplegia and autonomic dysreflexia, highlighting its potential as a viable rehabilitation strategy for patients with spinal cord injuries [11]. This underscores the broader applicability of BFR training in neurological rehabilitation, even in cases with complex medical conditions. Skiba et al. demonstrated that combining functional electro-stimulation with BFR significantly improved muscle mass and strength in individuals with spinal cord injuries, providing an effective strategy for counteracting muscle atrophy in affected areas [12]. This highlights the potential benefits of integrating BFR with other therapeutic modalities in spinal rehabilitation.

The prognosis for this patient is usually favorable as long the patient remains committed to maintaining a healthy lifestyle. Continued adherence to physical activity, a balanced diet, and attention to body mechanics are crucial in preventing the reoccurrence of symptoms. Periodic assessment can ensure that holistic management remains effective and that the patient does not experience any new symptoms. 

A limitation of this study is that post-treatment MRI was not conducted to assess structural changes in the spine. While MRI can provide detailed anatomical information and detect changes in disc protrusion or nerve compression, the focus of this study was on clinical outcomes such as pain reduction, body composition improvement, and functional recovery. Research has shown that MRI findings do not always correlate with clinical symptoms, and routine imaging may not be necessary if the patient demonstrates significant functional improvement and pain relief through conservative management [19]. Therefore, the decision to forego post-treatment MRI was made to emphasize the importance of functional recovery and patient-reported outcomes rather than anatomical findings alone.

## Conclusions

This study illustrates the feasibility and effectiveness of 12 weeks of BFR training in improving the body composition of a 26-year-old with L5-S1 disc protrusion with nerve root compression. The subject's weight decreased by 11.68 kg, while body mass index reduced by 3.65. TBFP decreased by 5.67%, and WHR reduced by 0.09. Significant improvements were also noted in the patient's maximal strength, indexed by 1RM, highlighting the potential benefits of BFR training in improving overall strength. While the BFR training showed significant benefits, it is important to acknowledge the combined effects of a calorie-deficit diet and regular exercise in achieving the observed outcomes. Healthcare providers may consider utilizing this treatment modality to improve physical fitness, muscle strength, and overall well-being in individuals with spinal injuries. Further research with large sample sizes and across diverse populations will help to understand the benefits of BFR training in spinal injuries.
